# Adjunct Diagnostic Accuracy of Antigen-specific Interleukin-2 for Latent Tuberculosis Infection in Pregnancy

**DOI:** 10.1093/ofid/ofag237

**Published:** 2026-04-25

**Authors:** Felix Bongomin, Phillip Ssekamatte, Ronald Olum, Diana Sitenda, Davis Kibirige, Joseph Baruch Baluku, Stephen Cose, Irene Andia-Biraro

**Affiliations:** Department of Medical Microbiology and Immunology, Faculty of Medicine, Gulu University, Gulu, Uganda; Department of Medical Microbiology and Immunology, Faculty of Medicine, Gulu University, Gulu, Uganda; Clinical Research Unit, Tuberculosis and Comorbidity Consortium, Kampala, Uganda; Department of Immunology and Molecular Biology, School of Biomedical Sciences, College of Health Sciences, Makerere University, Kampala, Uganda; Medical Research Council/Uganda Virus Research Institute (MRC/UVRI) and London School of Hygiene & Tropical Medicine (LSHTM) Uganda Research Unit, Entebbe, Uganda; Department of Epidemiology, Johns Hopkins Bloomberg School of Public Health, Baltimore, Maryland, USA; Clinical Research Unit, Tuberculosis and Comorbidity Consortium, Kampala, Uganda; Department of Immunology and Molecular Biology, School of Biomedical Sciences, College of Health Sciences, Makerere University, Kampala, Uganda; Clinical Research Unit, Tuberculosis and Comorbidity Consortium, Kampala, Uganda; Department of Medicine, Uganda Martyrs Lubaga Hospital, Kampala, Uganda; Makerere University Lung Institute, Kampala, Uganda; Division of Pulmonology, Kiruddu National Referral Hospital, Kampala, Uganda; Medical Research Council/Uganda Virus Research Institute (MRC/UVRI) and London School of Hygiene & Tropical Medicine (LSHTM) Uganda Research Unit, Entebbe, Uganda; Clinical Research Unit, Tuberculosis and Comorbidity Consortium, Kampala, Uganda; Department of Internal Medicine, School of Medicine, College of Health Sciences, Makerere University, Kampala, Uganda

**Keywords:** diagnostic biomarker, interleukin-2, latent tuberculosis infection, pregnant women

## Abstract

**Background:**

Pregnancy modulates immune responses against *Mycobacterium tuberculosis* (*Mtb*) infection. Cytokines, including interleukin-2 (IL-2), play a crucial role in *Mtb* containment, mainly by inducing the proliferation of T cells. However, the role of IL-2 in latent tuberculosis (TB) infection (LTBI) screening has not been completely explored. We, therefore, evaluated the diagnostic accuracy and optimal cutoff value (OCV) of IL-2 as a potential adjunct biomarker for screening LTBI during pregnancy.

**Methods:**

We enrolled pregnant women without previous active TB at Kawempe National Referral Hospital, Uganda, in 2020. We tested for LTBI using the QuantiFERON-TB Gold-Plus assay. Blood in quantiFERON blood collection tubes was stimulated with *Mtb* peptides of ESAT-6 and CFP-10 and following incubation, IL-2 culture supernatant levels were measured in an 11-plex Luminex assay using the Luminex® 100/200™ System with xPONENT® 3.1 software. LTBI positivity was defined as an IFN-γ concentration ≥0.35 IU/mL (calculated as TB1/TB2-Nil). A receiver operator characteristic curve was plotted to assess the diagnostic performance of *Mtb*-specific IL-2 culture supernatants using STATA 18.0. The OCV was determined using the Youden index, considering sensitivity and specificity.

**Results:**

Of 159 participants, the median gestational age was 26.1 (IQR: 20.0–31.6) weeks, 8.2% (n = 13) had HIV, and 10.1% (n = 16) were recent TB contacts. Overall, 79 (49.7%) had LTBI. *Mtb*-specific IL-2 levels were significantly higher in LTBI than in the non-LTBI group (847.9 (418.2–1309.1) versus 147.73 (100.38–207.19) pg/mL, *P* < .001). IL-2 achieved an AUC of 0.88 (95% CI: 0.82–0.93), with 76% sensitivity and 96% specificity. IFN-γ showed an AUC of 0.83 (95% CI: 0.76–0.90), with 78% sensitivity and 90% specificity. The combined IL-2 + IFN-γ response yielded the highest accuracy, with an AUC of 0.89 (95% CI: 0.83–0.94), 77% sensitivity, and 95% specificity.

**Conclusions:**

*Mtb*-specific IL-2 culture supernatant levels showed good discriminatory power for LTBI detection in pregnancy, comparable to IFN-γ, with the combined IL-2+ IFN-γ response yielding the highest accuracy. These findings support the potential utility of IL-2, alone or with IFN-γ, as an adjunct biomarker for LTBI screening in pregnant women, warranting further validation in larger, diverse cohorts.

## Teaser

IL-2, alone or in combination with IFN-γ, demonstrated good diagnostic accuracy for latent tuberculosis infection during pregnancy. These findings highlight IL-2 as a promising adjunct biomarker that could strengthen LTBI screening strategies in high-burden settings, including among women with HIV or recent TB exposure.

## Graphical Abstract

**Graphical Abstract:**
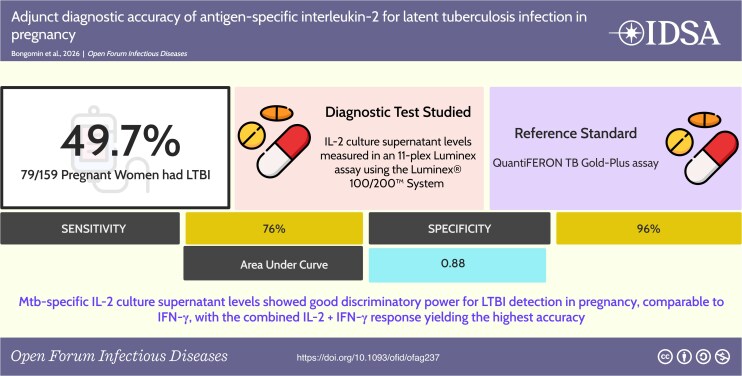
This graphical abstract is also available at Tidbit: https://tidbitapp.io/tidbits/adjunct-diagnostic-accuracy-of-antigen-specific-interleukin-2-for-latent-tuberculosis-infection-in-pregnancy?utm_campaign=tidbitlinkshare&=ITP

Latent tuberculosis infection (LTBI), an asymptomatic immunological state in which a person with *Mycobacterium tuberculosis* (*Mtb*) complex, was reported to affect about 24% of the world's population in 2019 [1]. LTBI is an important focus of tuberculosis (TB) control efforts because people with LTBI are at risk of developing active TB disease, and LTBI treatment can prevent progression to active TB disease [2]. The World Health Organization (WHO) Global TB report of 2024 reported that ∼10.8 million individuals worldwide were affected by TB in 2023 [3]. Of these cases, ∼3.4 million were women of reproductive age. TB contributes 6–15% to all maternal mortality [4]. However, a study estimated that over 200 000 annual cases of active TB disease occur during pregnancy globally [5].

TB in pregnancy poses a substantial risk of morbidity to both the pregnant woman and the fetus [4] if not diagnosed and treated in a timely manner. Immune dysregulation in pregnancy is associated with a more insidious onset of active TB, increased risk of LTBI and progression of LTBI to active TB disease [6]. Globally, an estimated 900 million women have a latent LTBI and have a considerably increased risk of reactivation to active disease during pregnancy or in puerperium [6]. Moreover, LTBI is associated with adverse pregnancy-related outcomes including abortions, still births, severe pre-eclampsia, low birth weight and the need for emergency cesarean section. In a recent systematic review, among pregnant women in the USA, the prevalence of LTBI ranged from 14 to 48% [7]. In this study, the diagnostic performance of both tuberculin skin test (TST) and interferon gamma release assay (IGRA) was comparable and was unaffected by pregnancy [7].

The WHO recommends testing and treatment of LTBI among high risk groups including infants and people living with HIV [8]. Pregnant women may be at increased risk of adverse pregnancy-related outcomes, partly due to physiological modulation of cellular immunity during gestation [9], which is central to controlling *Mtb* infection. Screening is currently performed using the TST or IGRA [10]. While a systematic review by Malhamé et al [7] found that the diagnostic performance of both tests was not significantly affected by pregnancy, biological evidence suggests that pregnancy-associated Th2 immune polarization may attenuate Th1-driven responses, including IFN-γ production, upon which the IGRA exclusively depends. Whether this translates to clinically meaningful reductions in IGRA sensitivity during pregnancy, particularly in the second and third trimesters and among immunocompromised women, remains an open and important research question. Both tests have limitations in pregnant women [11]. In their study, Tadjuddin et al., 2022 reported that the TST was more sensitive, 95% (95% confidence interval [CI]: 86.08%–98.96%) and less specific, 26.7% (95% CI: 12.28%–45.89%) compared to IGRA, however, the IGRA was more specific, 73.3% (95% CI: 54.11%–87.72%) and less sensitivity, 60% (95% CI: 46.54%–72.44%) compared to TST in screening for LTBI among pregnant women [11]. TST can produce false-positive results in individuals who have received the Bacille Calmette–Guérin (BCG) vaccine [12], which is commonly given in countries with high TB incidence. In addition, it has been reported that a few people given the TST return for reading [13], creating a gap in screening efforts. IGRA, on the other hand, is expensive and requires laboratory facilities and trained personnel, which skews the assay's feasibility toward research rather than as a point-of-care test in low-resource settings [14].

Interleukin-2 (IL-2) is a cytokine that plays a role in the immune response to *Mtb*, the bacterium that causes TB [15]. IL-2 has been studied as a potential diagnostic tool for LTBI [16, 17]. However, it has not been reported to have good sensitivity and specificity to stand alone as a biomarker that can distinguish LTBI from active TB [16].

Studies have reported the potential of IFN gamma, IL-2, and TNF cytokines in diagnosing LTBI, while recommending that the diagnostic accuracy of these cytokines should be further explored and validated [17–19]. Moreover, a combination of IFN gamma, IL-2, and TNF identified patients with pulmonary TB confirmed on culture with a negative Acid Fast Bacilli (AFB) microscopy smear from patients without pulmonary TB on both culture and the AFB microscopy smear [18]. A meta-analysis done by Wei et al. revealed that IL-2 cytokine (sensitivity/specificity: 0.87/0.61) showed the overall highest diagnostic potential as compared to IP-10 (0.77/0.73), IL-5 (0.64/0.75), IL-13 (0.75/0.71), IFN-γ (0.67/0.75), IL-10 (0.68/0.74), and TNF-α (0.67/0.64) [20]. IL-2 has also been reported to distinguish between IGRA-negative and IGRA-positive pregnant women in Indonesia [21]. The diagnostic performance of IP-10, IL-10, and IL-2 was assessed by the receiver operator curve (ROC) curve with the following AUC: 0.96 (*P* value <.001; 95% CI: 0.91–1.00), 0.67 (*P* value <.001; 95% CI: 0.57–0.76), and 0.65 (*P* value <.0132; 95% CI: 0.53–0.76), respectively [21]. We therefore evaluated the diagnostic accuracy of *Mtb*-specific IL-2 in a cohort of pregnant Ugandan women, exploring its potential as an adjunct biomarker to IFN-γ-based IGRA, particularly in the context of pregnancy-associated immune changes whose clinical significance for LTBI diagnostics has not yet been fully established.

## METHODS

### Study Design and Participants

Healthy pregnant women were enrolled into the study at Kawempe National Referral Hospital, Kampala, Uganda, between September 2020 and December 2020. Pregnant women who were willing to participate in the cross-sectional study regardless of gestational age or gravidity and those with known, suspected active TB or who had recently received TB treatment for the past 6 months were excluded. The study cohort, participant enrollment, and sample collection methods have been previously described in Bongomin et al. [22]. However, the cytokine measurements (including IL-2) and diagnostic accuracy analyses presented here have not been previously published and represent new findings from this cohort.

### Study Site

All laboratory procedures were performed at the immunology laboratory, Makerere University College of Health Sciences, Kampala, Uganda.

### Sample Size

This work is part of our previously published work [22], and our approach to sample size was as follows: using formula for a single population, we calculated a sample size of 260 participants based on an estimated prevalence of LTBI of 16.1% as reported in a previous study in the general population in Uganda [23], a margin of error of 5%, 20% incomplete data or withdrawal of consent, and a z statistic at a 95% CI.

### Laboratory Methods

#### Interferon Gamma Release Assay

The IGRA assay, QuantiFERON-TB Gold-plus (Qiagen, Hilden, Germany) was performed according to the manufacturer's instructions [24]. Briefly, about 1 mL of venous blood from 260 pregnant women was drawn directly into 4 QFT-plus tubes (Nil, TB1, TB2, Mitogen). The nil (negative control) tube contains no stimulants, the mitogen (positive control) tube contains phytohemagglutinin, the TB1 tube contains *Mtb*-specific peptides (ESAT-6 and CFP-10) modified for eliciting CD4^+^ T-cell responses, and the TB2 tube contains *Mtb*-specific peptides modified for eliciting both CD4^+^ and CD8^+^ T-cell responses. Tubes were placed in a 37°C incubator for 24 hours after being inverted 10 times for uniform mixing. Plasma was collected, and an enzyme-linked immunosorbent assay (ELISA) was performed to measure the amount of IFN-γ following the manufacturer's recommendations. Briefly, 50 µL of working-strength conjugate was added to the QFT-Plus ELISA plate, followed by 50 µL of the plasma and standards to the appropriate wells. The plate was incubated for 2 hours, after which they were washed 6 times with 400 µL of 1× wash buffer. 100 µL of the substrate solution was added, and the plate was incubated for 30 minutes in the dark. This was followed by the addition of 50 µL of stop solution immediately. The plate was immediately read using an ELISA reader at 450 nm with a reference wavelength of 620 nm to obtain optical densities. Results were calculated using QFT-Plus analysis software version 2.71.2. LTBI positivity was defined as an IFN-γ concentration ≥0.35 IU/mL (calculated as TB1/TB2-Nil). If TB1/TB2-Nil was <0.35 IU/mL or <25% of the Nil value, when the mitogen was ≥0.5 IU/mL, the result was considered negative per the manufacturer's guidelines [24].

#### Luminex Assay

A total of 162 TB2 tube ESAT-6 and CFP-10 stimulated plasma samples were thawed, randomized and analyzed to determine the concentration of 11 analytes IL-6, IL-2, c-reactive protein (CRP), interferon (IFN)-γ, IL-4, IL-5, IFN-α, IL-10, IL-17/IL-17A, macrophage inflammatory protein (MIP)-1α, and MIP-1β. The samples were undiluted and represented 2 patient groups (LTBI positive and LTBI negative). Briefly, all samples and Luminex kits were thawed at room temperature for 1 hour; 50 µl of sample, standards, and Calibrator diluent (for the blank wells) were loaded into the selected wells. Next, 50 µl of reconstituted microparticle cocktail to all wells, followed by the incubation of the plates at room temperature for 2 hours on a horizontal orbital plate shaker at 800 rpm in the dark. The plates were washed 3 times with 100 µl of 1× wash buffer using a Luminex magnetic plate, followed by the addition of 50 µl of reconstituted biotin-antibody cocktail to all wells, incubated for 1 hour at room temperature, on a horizontal orbital plate shaker at 800 rpm in the dark. The plates were rewashed, 50 µl of diluted streptavidin-PE was added to all wells, and the incubation procedure was repeated for 30 minutes. The plates were washed, and finally, 100 µl of wash buffer was added to all wells and incubated for 2 minutes at room temperature on a shaker at 800 rpm in the dark. The plates were then placed in the Milliplex luminex 100/200™ analyser with xPONENT® program (version 3.1 Merck, Millipore) to determine the bead count, mean fluorescence intensity, and concentrations of the *Mtb*-specific cytokines in the samples using the Luminex xPONENT® program version 3.1 (Merck, Millipore). The concentration values and detection limits were determined and extrapolated from standard curves generated from each kit's specific standards using the xPONENT® weighted 5PL curve fitting procedure.

### Statistical Analysis

Data analysis was performed in STATA 18.0 and R (version 4.4.2; R Foundation for Statistical Computing, Vienna, Austria). Baseline characteristics were summarized as median (IQR) for continuous variables and as frequency (percentages) for categorical variables. The differences in cytokine levels by LTBI status were evaluated using a Mann–Whitney *U* test and a ROC plotted to assess its diagnostic performance, reporting the area under the curve and ROC curves. Further analysis was performed to evaluate cytokines that showed a significant association with LTBI. First, we compared their performance by HIV status and history of TB contact. We then performed logistic regression analysis to adjust for potential confounders measured (age, gestational age, adjusted body mass index, HIV status, HbA1c levels, and previous alcohol use), from the review of the literature. A *P* < .05 was considered statistically significant. The optimal cutoff for the cytokines and the diagnostic performance at this cutoff (sensitivity, specificity) was identified using the Youden Index method (cutpt command in Stata 18.0).

### Ethical Statement

Study participants provided informed written consent after the study procedure, risks, and benefits were explained to them. The study protocol was approved by the Makerere University School of Medicine Ethics and Research Committee (reference number 2020-113). All principles of research involving human subjects outlined in the *Declaration of Helsinki* were adhered to.

## RESULTS

### Participant Demographics

Out of 260 participants, 162 participant samples were assayed, and of these, data for 159 pregnant women with complete cytokine results were included. Of these, 80 (50.3%) were LTBI positive and 79 (49.7%) LTBI negative. The median age was 27.0 years (IQR: 24.0–32.0) with a median gestational age of 26.1 weeks (IQR: 20.0–31.6). Only 8.2% (n = 13) were living with HIV and 10.1% (n = 16) had a recent contact with a person living with TB. The median HbA1c levels were 5.1% (IQR: 4.8–5.6), with 19.5% (n = 31) and 1.9% (n = 3) having prediabetes and diabetes, respectively. Table 1 summarizes the baseline characteristics.

**Table 1.** Demographic Characteristics

Variable	Overall	No LTBI	LTBI	*P* Value*^a^*
	N = 159*^b^*	N = 80*^b^*	N = 79*^b^*	
Age: median/IQR	27.0 (24.0, 32.0)	26.0 (23.0, 30.0)	29.0 (24.0, 32.0)	.035
Gestational age in weeks	26 (20, 32)	25 (19, 31)	27 (20, 32)	.540
Adjusted body mass index	27.2 (23.3, 31.2)	27.3 (23.3, 31.6)	27.2 (23.3, 29.4)	.454
BMI categories				.313
Underweight	50 (31%)	26 (33%)	24 (30%)	
Normal	45 (28%)	27 (34%)	18 (23%)	
Overweight	61 (38%)	26 (33%)	35 (44%)	
Obesity	3 (1.9%)	1 (1.3%)	2 (2.5%)	
Alcohol use				.033
Never	25 (16%)	18 (23%)	7 (8.9%)	
Former	35 (22%)	19 (24%)	16 (20%)	
Current	99 (62%)	43 (54%)	56 (71%)	
Smoking history				>.999
Former	1 (0.6%)	1 (1.3%)	0 (0%)	
Never	158 (99%)	79 (99%)	79 (100%)	
HIV status				.141
Negative	146 (92%)	76 (95%)	70 (89%)	
Positive	13 (8.2%)	4 (5.0%)	9 (11%)	
BCG scar				.285
Present	52 (33%)	23 (29%)	29 (37%)	
Absent	107 (67%)	57 (71%)	50 (63%)	
Family history of TB				.233
Positive	140 (88%)	68 (85%)	72 (91%)	
Negative	19 (12%)	12 (15%)	7 (8.9%)	
Recent TB contact	16 (10%)	12 (15%)	4 (5.1%)	.037

Bold = statistically significant.

^*a*^Wilcoxon rank sum test; Fisher's exact test; Pearson's χ^2^ test.

^*b*^Median (Q1, Q3); n (%).

### LTBI-positive Pregnant Women Expressed Higher Levels of IL-2 in Their QFT-tube Culture Supernatants Compared to LTBI-negative Women

We did not observe any differences in the cytokine levels for LTBI-negative and LTBI-positive pregnant women for the following markers: IL-6, IL-10, MIP-1 alpha, MIP-1 beta, IL-4, IL-17/17A, IL-5, CRP, and IFN-α (Tables 2 and 3).

**Table 2.** Median Concentration of Cytokine Production in LTBI and Non-LTBI Groups of Pregnant Women

Characteristic	Overall, N = 159*^a^*	No LTBI, N = 80*^a^*	LTBI, N = 79*^a^*	*P* Value*^b^*	ROC AUC (95% CI)
IL-6	1537 (754–2302)	1561 (715–2285)	1434 (755–2330)	.900	0.506 (0.415–0.597)
IL-10	32 (21–47)	33 (19–52)	29 (21–43)	.382	0.460 (0.369–0.551)
IFN-gamma	369 (248–1000)	369 (212–369)	963 (464–1409)	**<**.**001**	0.832 (0.764–0.900)
MIP-1 alpha	8063 (5134–12 306)	8305 (4402–12 529)	7716 (5612–12 306)	.922	0.505 (0.413–0.596)
MIP-1 beta	7805 (5084–10 114)	7224 (4926–9868)	8022 (5701–10 836)	.072	0.583 (0.494–0.672)
IL-4	260 (218–295)	249 (218–292)	264 (229–306)	.175	0.563 (0.472–0.653)
IL-17/IL-17A	74 (53–94)	64 (53–94)	74 (53–94)	.133	0.568 (0.480–0.656)
IL-2	207 (133–874)	148 (100–207)	848 (418–1309)	**<**.**001**	0.878 (0.820–0.935)
IL-5	55.63 (55.63–55.63)	55.63 (55.63–55.63)	55.63 (55.63–55.63)	.802	0.506 (0.459–0.552)
CRP	7254 (5368–11 376)	7437 (5407–13 069)	7127 (5332–11 207)	.710	0.483 (0.392–0.573)
IFN-alpha	49 (38–73)	46 (35–73)	50 (39–73)	.196	0.560 (0.469–0.652)

Bold = statistically significant.

^*a*^Median (Q1, Q3).

^*b*^Wilcoxon rank sum test.

**Table 3.** Diagnostic Performance and Optimal Cutoff Level of 6 Cytokines in Discriminating Pregnant Women With and Without Latent TB Infection

Cytokine	Optimal Cutoff point (pg/mL)	Youden Index	Sensitivity (%)	Specificity (%)	AUC at Cut-point
IL2	389.2	0.722	76	96	0.86
IL-17/IL-17A	65.1	0.145	63	51	0.57
IFN-alpha	49.8	0.135	53	61	0.57
IFN-gamma	405.2	0.685	78	90	0.84
IL6	589.4	0.101	86	24	0.55
IL5	101.6	0.063	06	100	0.53
CRP	4389.3	0.061	91	15	0.53

IL-2 levels were statistically significantly higher among LTBI-positive [847.9 (418.208–1309.09)] compared to LTBI-negative [147.73 (100.38–207.19)] women, *P* < .001. A unit increase in IL-2 levels was significantly associated with a 0.3% increase in the odds of having LTBI (crude OR: 1.003, 95% CI: 1.002–1.004, *P* < .001), even after adjusting for potential confounders (adjusted OR: 1.003, 95% CI: 1.002–1.005, *P* < .001) (Table 4).

**Table 4.** Adjusted Odds Ratio for IL-2 Levels and Demographic Characteristics Among Pregnant Women

Variable	Adjusted Odds Ratio	95% CI	*P* Value
IL-2	1.007	1.004–1.010	**<**.**001**
IFN gamma	0.997	0.996–0.999	.**003**
Mother's age in years	1.043	0.963–1.131	.301
Gestational age in weeks	0.979	0.926–1.035	.458
HBA1c (%)	0.873	0.573–1.331	.528
HIV status			
Negative	Reference		
Positive	2.333	0.490–11.100	.287
Adjusted body mass index			
Normal	Reference		
Underweight	0.123	0.003–4.325	.248
Overweight	0.395	0.012–13.213	.604
Obese	0.128	0.004–4.535	.259
Alcohol use			
Never	Reference		
Former	0.342	0.104–1.128	.078
Current	0.333	0.094–1.173	.087
TB contact			
No	Reference		
Yes	0.223	0.045–1.096	.065

### IL-2 Showed Good Diagnostic Performance of LTBI Screening Among Pregnant Women Living With HIV and TB Contacts

**Figure 1.**
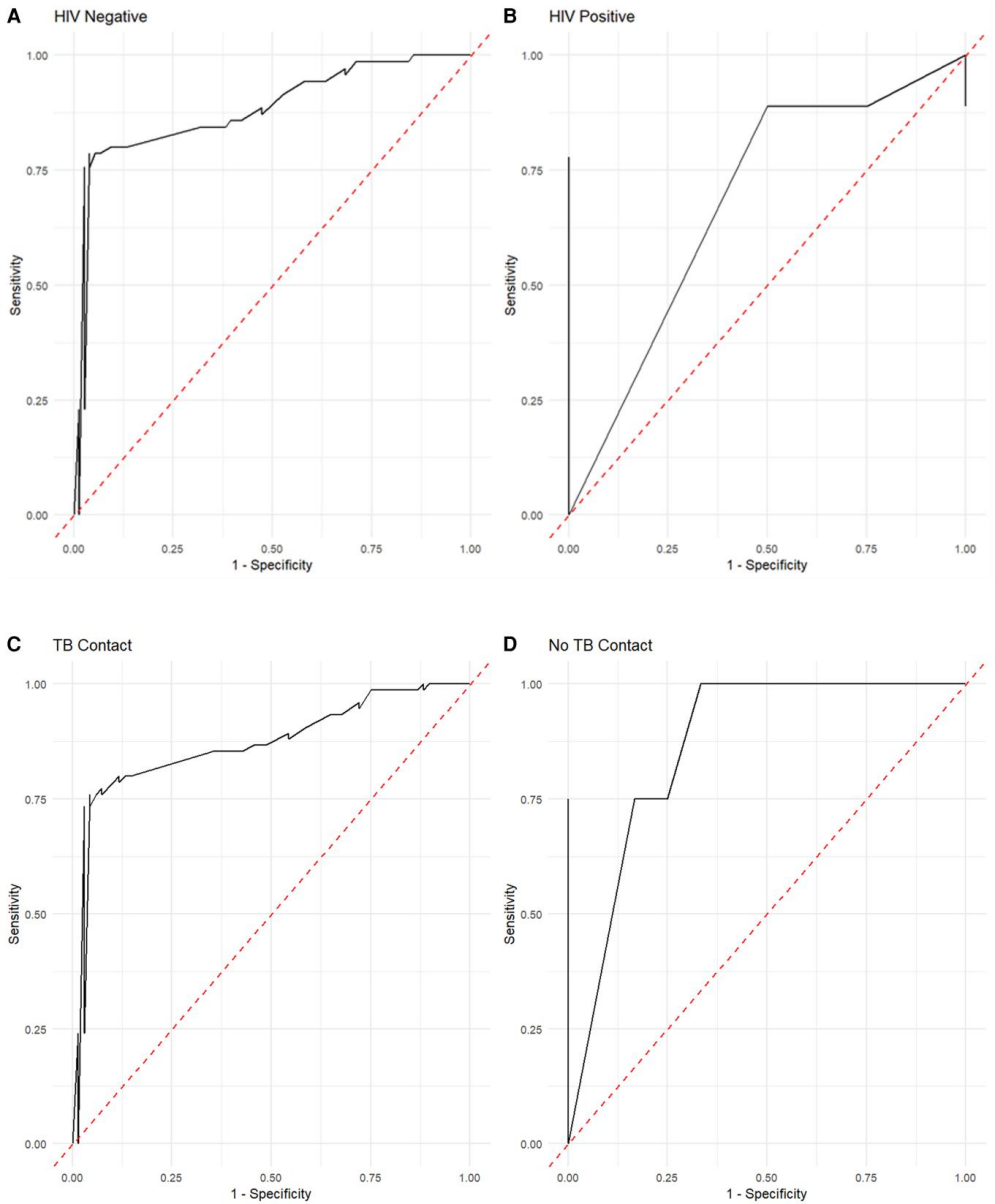
Receiver operator characteristic curves to demonstrate the diagnostic potential of IL-2 among pregnant women living without HIV (Panel *A*), with HIV (Panel *B*), with a history of TB contact (Panel *C*), and without a history of TB contact.

In diagnosing LTBI among HIV-positive cases, at an optimal cutoff point of 247.2, IL-2 offered a sensitivity of 78%, and a specificity of 100%, with an AUC of 0.89 (95% CI: 0.641–1.000) as shown in Figure 1*B*. In addition, IL-2 discriminated for LTBI among HIV-negative cases, at an optimal cutoff point of 389.2, with a sensitivity of 79%, a specificity of 96%, and an AUC of 0.87 (95% CI: 0.822–0.940), Figure 1*A*. For TB contacts, IL-2, at an optimal cutoff point of 384.7, discriminated for LTBI with a sensitivity of 75%, a specificity of 100%, and an AUC of 0.88 (95% CI: 0.771–1.000) as shown in Figure 1*C*. IL-2 finally discriminated for LTBI among non-TB contacts, at an optimal cutoff point of 389.2, with a sensitivity of 76%, a specificity of 96%, and an AUC of 0.86 (95% CI: 0.807–0.932) (Figure 1*D*).

**Figure 2.**
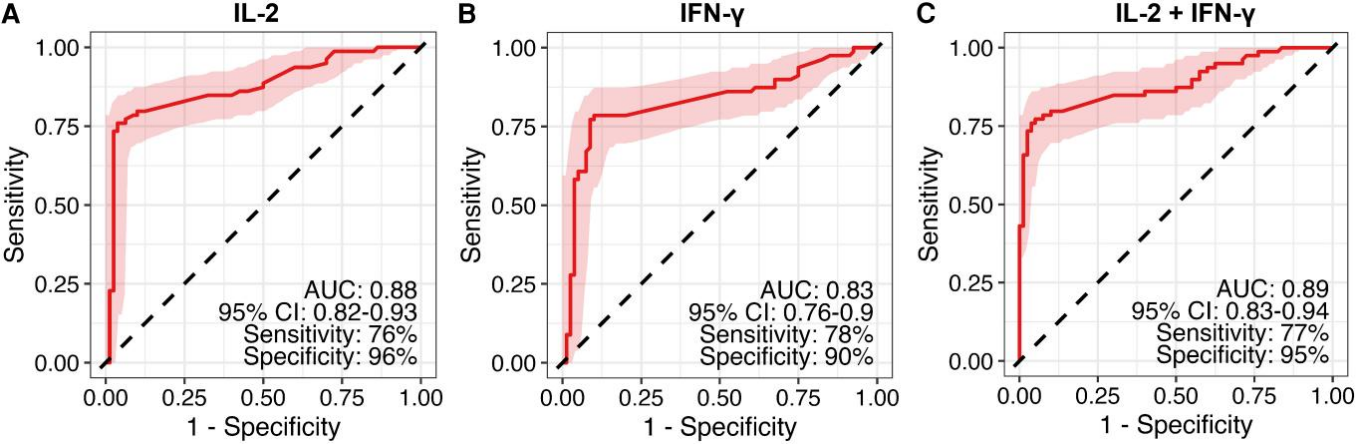
Receiver operator characteristic curves to demonstrate the diagnostic potential of IL-2 alone (Panel *A*), interferon gamma alone (Panel *B*), and IL-2 in combination with interferon gamma (Panel *C*) for the diagnosis of latent TB infection in pregnancy.

IL-2 achieved an AUC of 0.88 (95% CI: 0.82–0.93), with 76% sensitivity and 96% specificity. IFN-γ showed an AUC of 0.83 (95% CI: 0.76–0.90), with 78% sensitivity and 90% specificity. The combined IL-2 + IFN-γ response yielded the highest accuracy, with an AUC of 0.89 (95% CI: 0.83–0.94), 77% sensitivity, and 95% specificity, Figure 2.

## DISCUSSION

LTBI is impeding end TB goals because the existing screening tests have their shortcomings, resulting in some people not benefiting from screening and ultimately, causing progression to TB, which could have been avoided [25]. We evaluated the diagnostic performance of IL-2 for LTBI screening among pregnant women, based on the fact that pregnancy physiological changes have been reported to influence the Th1 immunity [26], resulting in reduced reliability on markers like IFN-γ, which is solely detected by the existing IGRA, for testing LTBI. The levels of IFN-γ are reported to be lower during the second and third trimesters of pregnancy, thus making the IGRA an unreliable test during this period [27]. In this study, we report 3 main findings: (1) LTBI-positive pregnant women had higher plasma IL-2 levels compared with LTBI-negative women; (2) IL-2 showed good diagnostic accuracy, with an AUC of 0.88 (95% CI: 0.82–0.93), sensitivity of 76%, and specificity of 96%, while IFN-γ also performed well (AUC = 0.83, 95% CI: 0.76–0.90; sensitivity 78%, specificity 90%); and (3) the combined IL-2 + IFN-γ response provided the best overall performance (AUC = 0.89, 95% CI: 0.83–0.94; sensitivity 77%, specificity 95%), with consistent diagnostic value across subgroups including women with and without HIV infection, and both TB contacts and noncontacts.

Previous studies have reported the potential of IL-2 as a biomarker for LTBI [17, 19, 28, 29]. Moreover, Sun et al. reported the potential of a combination of IL-2 and IFN gamma to discriminate between LTBI and active TB based on ROC curve analysis [30]. The observed high diagnostic performance of IL-2 holds potential for its utility in LTBI screening, particularly in populations where conventional tests may have limitations, for example, pregnant women and those with diabetes. Pregnancy induces immunological changes that can affect the performance of traditional LTBI diagnostics such as TST and IGRAs. The immunosuppressive state during pregnancy, characterized by a shift toward a Th2-dominant response [31], can diminish the sensitivity of these tests. LaCourse et al. reported reduced mitogen in pregnant women compared to women in the postpartum stage, suggesting that pregnancy can influence results from the IGRA [32]. In contrast, IL-2 production, associated with Th1 responses, may remain relatively stable, making it a reliable marker during pregnancy.

Our study's observation that IL-2 maintains diagnostic performance irrespective of HIV status is relevant given that HIV infection impairs cell-mediated immunity [33], often leading to false-negative results in TST and IGRAs [34, 35]. However, IL-2 levels were consistently elevated in LTBI-positive individuals regardless of HIV status, suggesting its robustness as a diagnostic marker in immunocompromised populations. Furthermore, the ability of IL-2 to discriminate LTBI status among both TB contacts and noncontacts indicates its potential in broader screening programs. This is crucial in high TB-burden settings like Uganda, where identifying and treating LTBI is essential for TB control [36]. While IL-2 shows promise, other cytokines like interferon gamma-induced protein 10 (IP-10) have also been explored as potential biomarkers for LTBI. Some studies suggest that IP-10 may offer higher diagnostic performance compared to IL-2 [37]. However, the choice of biomarker may depend on specific population characteristics, like in this case, pregnancy.

Despite these promising findings, we acknowledge the following limitations. First, this was a cross-sectional design and therefore, IL-2 levels were not assessed over time, yet biomarker expression is reported to be dependent on the stage of a pregnancy (gestation age) [38]. Secondly, the measurement of IL-2 relies on the cultured plasma stimulated by the QFT-blood collection tubes, *Mtb* peptides of ESAT-6 and CFP-10. A lateral flow assay could, however, be developed whereby strips are used to test for LTBI, or a new assay measuring IL-2, could be developed to enhance screening for LTBI in vulnerable groups, including pregnant women. Thirdly, the combined IL-2 and IFN-γ diagnostic model was derived and evaluated within the same dataset without external validation, introducing potential bias in the reported performance metrics. Lastly, a fundamental limitation of this study is the use of QFT-plus as both the reference standard for LTBI classification and the comparator for IL-2 diagnostic performance. This circular framework cannot, by design, demonstrate whether IL-2 detects true *Mtb* sensitization in individuals scored negative by QFT-plus.

## CONCLUSIONS

At an optimal cutoff point of 389.2, IL-2 demonstrated moderate sensitivity (76%) and high specificity (96%) with good discriminatory power. IFN-γ showed comparable performance with an AUC of 0.83, sensitivity of 78%, and specificity of 90%. Notably, combining IL-2 and IFN-γ yielded the best overall diagnostic accuracy (AUC = 0.89) with balanced sensitivity (77%) and specificity (95%). These findings indicate that IL-2, alone or in combination with IFN-γ, provides reliable discriminatory power for detecting LTBI among pregnant women, with performance metrics similar to those reported in the general population. Given the high TB burden in low-resource settings, considerations of cost, feasibility, and accessibility are critical when implementing IL-2-based assays. Moreover, head-to-head comparisons with existing diagnostic tools such as TST and IGRA are necessary to establish their relative strengths and limitations. This study supports the potential utility of IL-2, particularly in combination with IFN-γ, as a diagnostic biomarker for LTBI in pregnant women, including those with HIV or varied TB exposure histories. Further longitudinal studies are warranted to validate these findings and evaluate integration into TB control program.
